# Sprouty3, but Not Sprouty1, Expression Is Beneficial for the Malignant Potential of Osteosarcoma Cells

**DOI:** 10.3390/ijms222111944

**Published:** 2021-11-04

**Authors:** Anna Zita Mehira Kamptner, Christoph-Erik Mayer, Hedwig Sutterlüty

**Affiliations:** Institute of Cancer Research, Department of Medicine I, Comprehensive Cancer Center, Medical University of Vienna, A-1090 Vienna, Austria; anna.kamptner@gmx.net (A.Z.M.K.); ce.mayer@gmx.at (C.-E.M.)

**Keywords:** Sprouty3, osteosarcoma, Spry3, Spry1, tumor promoter, MAPK pathway, ERK signaling, RTK modulator

## Abstract

Sprouty proteins are widely accepted modulators of receptor tyrosine kinase-associated pathways and fulfill diversified roles in cancerogenesis dependent on the originating cells. In this study we detected a high expression of Sprouty3 in osteosarcoma-derived cells and addressed the question of whether Sprouty3 and Sprouty1 influence the malignant phenotype of this bone tumor entity. By using adenoviruses, the Sprouty proteins were expressed in two different cell lines and their influence on cellular behavior was assessed. Growth curve analyses and Scratch assays revealed that Sprouty3 accelerates cell proliferation and migration. Additionally, more colonies were grown in Soft agar if the cells express Sprouty3. In parallel, Sprouty1 had no significant effect on the measured endpoints of the study in osteosarcoma-derived cells. The promotion of the tumorigenic capacities in the presence of Sprouty3 coincided with an increased activation of signaling as measured by evaluating the phosphorylation of extracellular signal-regulated kinases (ERKs). Ectopic expression of a mutated Sprouty3 protein, in which the tyrosine necessary for its activation was substituted, resulted in inhibited migration of the treated cells. Our findings identify Sprouty3 as a candidate for a tumor promoter in osteosarcoma.

## 1. Introduction

Osteosarcoma is the most common malignant tumor of bones. It shows a biphasic age profile by mainly affecting adolescents and individuals over age 60 [1,2]. Although the five-year-survival-rate is around 70% if treatment is applied, this rate has remained unchanged for the past several decades [3]. The development of osteosarcomas is not connected to common specific genetic aberrations, but uncontrolled cell proliferation is a crucial step in their cancerogenesis [4].

Cellular processes such as proliferation, migration, differentiation, growth, and cell survival are strictly controlled by intercellular communication via receptor tyrosine kinases (RTKs). Stimulation of the mitogen-activated protein kinase (MAPK) cascade, the phospholipase Cγ (PLCγ), the lipid kinase phosphatidylinositol-3-kinase (PI3K)/Akt, and the signal transducer and activator of transcription (STAT) pathway converts the signals received by these receptors to cellular responses reflected by different expression profiles [5].

Fine tuning of the RTK-induced transduction pathways is crucial for the cellular interpretation of extracellular cues [6]. In these regulative processes, Sprouty (Spry) proteins play an important role by orchestrating between different signaling pathways.

In mammals, four Spry homologues have been identified [7]. Knockout experiments in mice revealed that concomitant loss of Spry2 and Spry4 is lethal [8], while the lack of only Spry1 [9,10], Spry2 [11,12] or Spry4 [8] produces viable mice with specific phenotypes indicating that the functional compensation of the other Spry members is only partly.

Further analyses revealed that Spry proteins function mainly by specifically interfering with the MAPK-pathway at different levels downstream of diverse RTKs [13,14,15]. Additionally, inhibition of signal transduction via the PI3K/Akt [16] and PLCγ pathway [17] was reported occasionally. In contrast to these capacities as negative regulatory effectors of signal transduction, Spry proteins are endowed with properties resulting in intensified signaling. An N-terminal SH2 interacting motif, which comprises an evolutionarily conserved tyrosine residue, can function as canonical casitas B-lineage lymphoma (c-Cbl) binding domain and thereby Spry proteins interfere with receptor degradation processes [18,19].

Considering their importance in regulating signal transduction and the common involvement of altered RTK-mediated pathways in cancerogenesis, multiple studies investigated the role of Spry proteins in diverse tumors [20]. Spry2 is frequently found to function as tumor suppressor in cancers of e.g., lung [21,22], breast [23], and liver [24]), but in other tumor entities it exerts tumor promoting tasks (e.g., in colon [25] and brain [26,27] cancers). Additionally, Spry4 is a well-documented tumor suppressor in tumors originating from e.g., lung [28], breast [29], and brain [30]. Spry1 promotes the tumorigenic capacities of rhabdomyosarcoma [31], while this member of the Spry protein family exerts tumor-suppressive roles in cancers of the prostate [32] and thyroid [33]. In contrast, the role of Spry3 is only studied in glioblastoma, where it is like Spry2 [26,27] functioning as tumor promoter [30].

In the presented study we analyzed if Spry3 expression is a unique feature of glioblastoma and found that Spry3 is well expressed in osteosarcoma. Therefore we studied how its expression is influencing osteosarcoma-derived cells. The influence of Spry2 and Spry4 on the malignant phenotype of osteosarcoma is already investigated [34]. These studies show that Spry2 is functioning as efficient tumor suppressor in osteosarcoma [34]. To complete the studies concerning the different roles of Spry proteins in this tumor entity, Spry1 was additionally included in the study.

## 2. Results

### 2.1. Osteosarcoma-Derived Cell Lines Express Substantial Levels of Spry3 Protein

**Figure 1:**
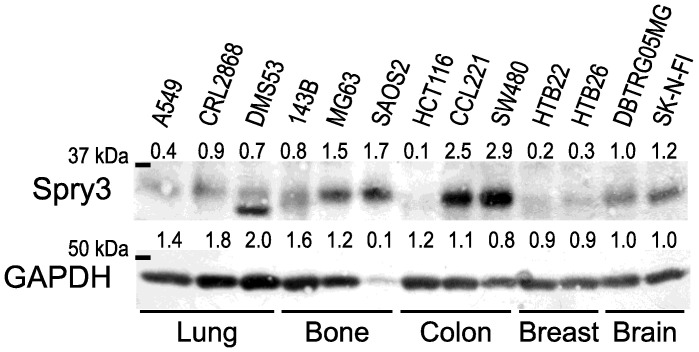
Expression of Spry3 protein in cancer-derived cell lines. An immunoblot of logarithmically growing cells of the indicated cell lines derived from lung, bone, colon, breast and brain cancers was performed and sequentially probed with antibodies recognizing Spry3 and GAPDH, respectively. Quantification of the immunoblot was performed using ImageQuant software (Molecular Dynamics, Sunnyvale, CA, USA). DBTRG05MG was arbitrarily set as 1.

Earlier studies indicate that Spry3 expression in adult mammalians is restricted to the testis and brain [7]. To evaluate if Spry3 is expressed in cancer cells originating from different tissues, we first compared Spry3 expression in cell lines derived from lung, bone, colon and breast tumors with the levels observed in brain cancer-derived cell lines. Immunoblotting of logarithmically growing cells revealed that in contrast to breast cancer-derived cell lines, cells originated from osteosarcoma express prominent levels of Spry3 exceeding the ones observed in the brain cancer cell lines (Figure 1). In the tested lung cancer-derived cell lines the detected Spry3 levels were comparably low, while in colon cancer Spry3 was clearly detected in two of the three tested cell lines (Figure 1).

These data indicate that Spry3 expression is very abundant in osteosarcoma, indicating that it could play a role in this tumor entity.

### 2.2. Irrespective of Mitogen Availability, Spry3 and Spry1 Proteins Are Usually Expressed in Osteosarcoma

**Figure 2:**
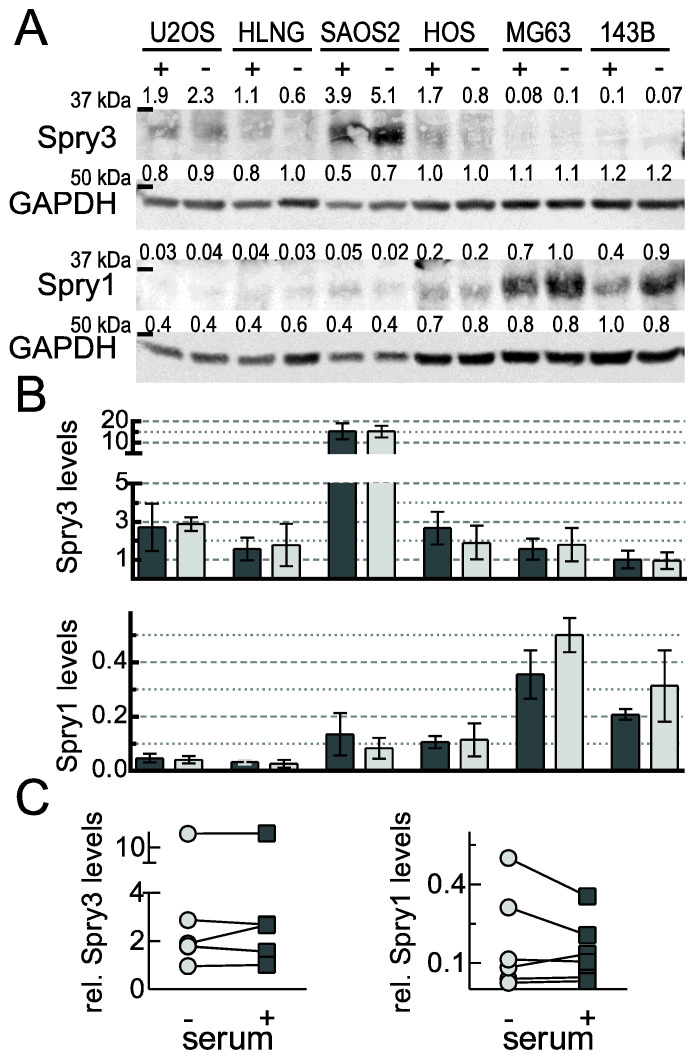
Endogenous Spry3 and Spry1 protein levels in osteosarcoma-derived cell lines. (**A**) The indicated osteosarcoma-derived cell lines were cultured for 24 h in the absence (−) or presence (+) of serum. Using immunoblotting, endogenous Spry3 and Spry1 protein amounts were detected. Loading was determined by using antibodies recognizing GAPDH. The mean values were arbitrarily set as 1. (**B**) Quantification results of three independent performed Western blot experiments are depicted as mean ± SEM in a column graph. Expression levels of Spry3 and Spry1 proteins were calculated as the ratio to the mean values of the experiment (in case of Spry3 the numbers for SAOS2 were not included) by Image Quant software and normalized to GAPDH. (**C**) Quantified Spry3 and Spry1 levels from cells grown in serum-deprived (circle) and –supplemented (square) mediums are compared.

In the following experiments we characterized six osteosarcoma-derived cell lines concerning their relative expression levels of Spry3 and Spry1 and evaluated how mitogen availability is influencing these expression levels. Therefore, 60% confluent cells were cultivated for another 24 h in absence (−) or presence (+) of serum. As depicted in Figure 2, all osteosarcoma-derived cell lines express Spry3 and Spry1, but wide variations were detected. Although the two cell lines expressing high levels of Spry1 (MG63 and 143B) show rather low levels of Spry3 (Figure 2A,B), neither a significant negative nor a positive correlation could be calculated. The serum availability in the cellular environment had no influence on the expression of either Spry3 or Spry1 (Figure 2C).

### 2.3. In Osteosarcoma-Derived Cells, Spry3, but Not Spry1, Accelerates Cell Proliferation

**Figure 3:**
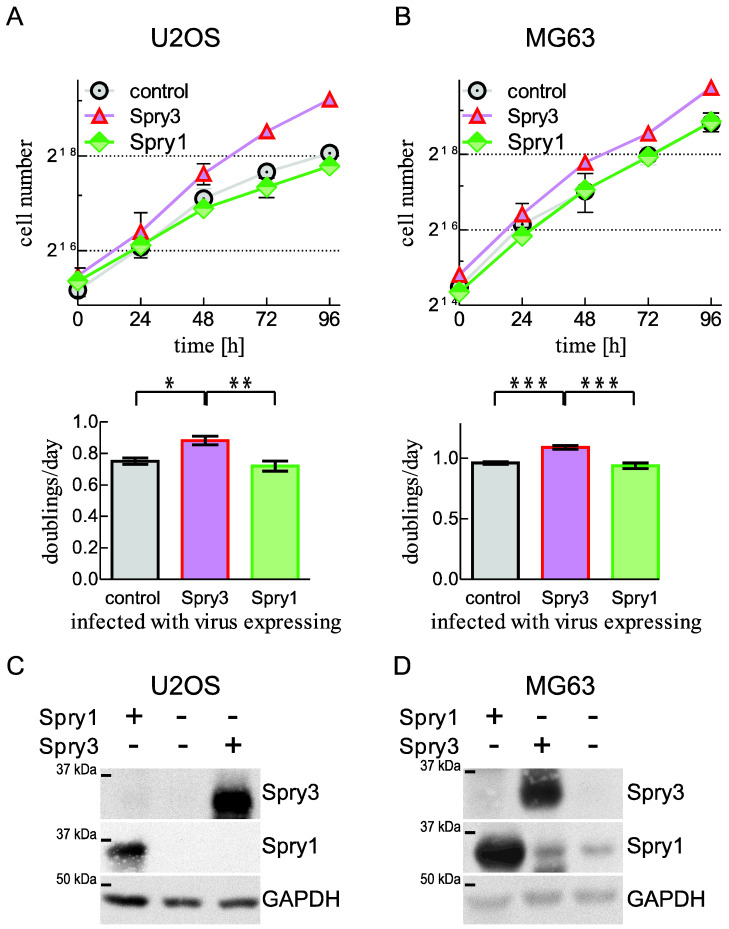
Influence of Spry3 and Spry1 expression on cell proliferation of osteosarcoma-derived cells. U2OS (**A**) and MG63 (**B**) cells infected with adenoviruses expressing Spry3, Spry1 or a control protein were analyzed. The number of cells were counted every 24 h for four days. A representative growth curve depicting the counted cell number of three replicates using a log2 scale is shown. Using GraphPad Prism 5.0 software (San Diego, CA, USA), doubling times of at least three independent growth curve analyses were calculated using exponential equation. The calculated values are presented as mean doublings per day ± SEM. Overexpressions of Spry1 and Spry3 in U2OS (**C**) and MG63 (**D**) were verified by immunoblotting using the indicated antibodies. *** p < 0.001, ** p < 0.01; * p < 0.05.

Tumor formation is commonly considered as consequence of excessive growth mainly caused by increased cell proliferation. Therefore, we first evaluated if elevated Spry1 and Spry3 levels are able to influence the cell proliferation of osteosarcoma-derived cells. U2OS cells express hardly detectable amounts of Spry1, while the Spry3 amounts are more pronounced than in the most other osteosarcoma-derived cell lines, but about five times less than in SAOS2 cells. Furthermore, this cell line is well susceptible to adenoviral infection (data not shown and [34]). Additionally, these cancer cells are known to show an intact response to mitogen supplementation [34]. As depicted in Figure 3A, compared to cells expressing luciferase, which was used as a control protein, the number of Spry3 expressing cells accumulated progressively within the 4 day study time. Calculation of doubling times using exponential growth equations revealed that Spry3 expression accelerated the cell proliferation to 0.88 ± 0.03 as compared to 0.75 ± 0.02 doublings per day (Figure 3A lower panel). Cell proliferation in case of Spry1 expression was within the fluctuations of the growth curves obtained for control-treated U2OS (Figure 3A). To study if the effect of the investigated Spry protein also applies to other osteosarcoma-derived cell lines, growth curve analyses were performed in MG63 cells. In contrast to U2OS cells, which express rather high Spry3 and low Spry1 levels, MG63 is the osteosarcoma-derived cell line with the highest Spry1 levels and has rather low Spry3 levels. Additionally, this cell line is derived from a tumor in a male patient, while U2OS were established from an osteosarcoma of female origin. Since the Spry3 encoding gene is localized in the pseudoautosomal region, gender-specific roles cannot be ruled out. Despite the differences, expressions of both Spry proteins show the same effect on proliferation as observed in U2OS cells (Figure 3B). Elevation of Spry1 protein levels had no influence on the proliferative capacity of the MG63 cell line, while the presence of Spry3 causes accelerated growth (Figure 3B). Cells with increased Spry3 levels double more than once per day, while control treated cells perform only 0.95 doublings per 24 h (Figure 3B lower panel). Like in U2OS cells, Spry1 protein levels fail to significantly affect cell proliferation.

In U2OS (Figure 3C) as well as in MG63 (Figure 3D), both infection with Spry3 encoding and Spry1 harboring viruses results in a pronounced overexpression of the protein.

These data indicate that expression of Spry3 has a promoting effect on cell proliferation of different osteosarcoma-derived cell lines, while Spry1 protein levels are insubstantial for this cellular process.

### 2.4. A Tumor Promoting Role of Spry3 Is Additionally Suggested by Its Beneficial Role on Cell Migration of Osteosarcoma-Derived Cells

**Figure 4:**
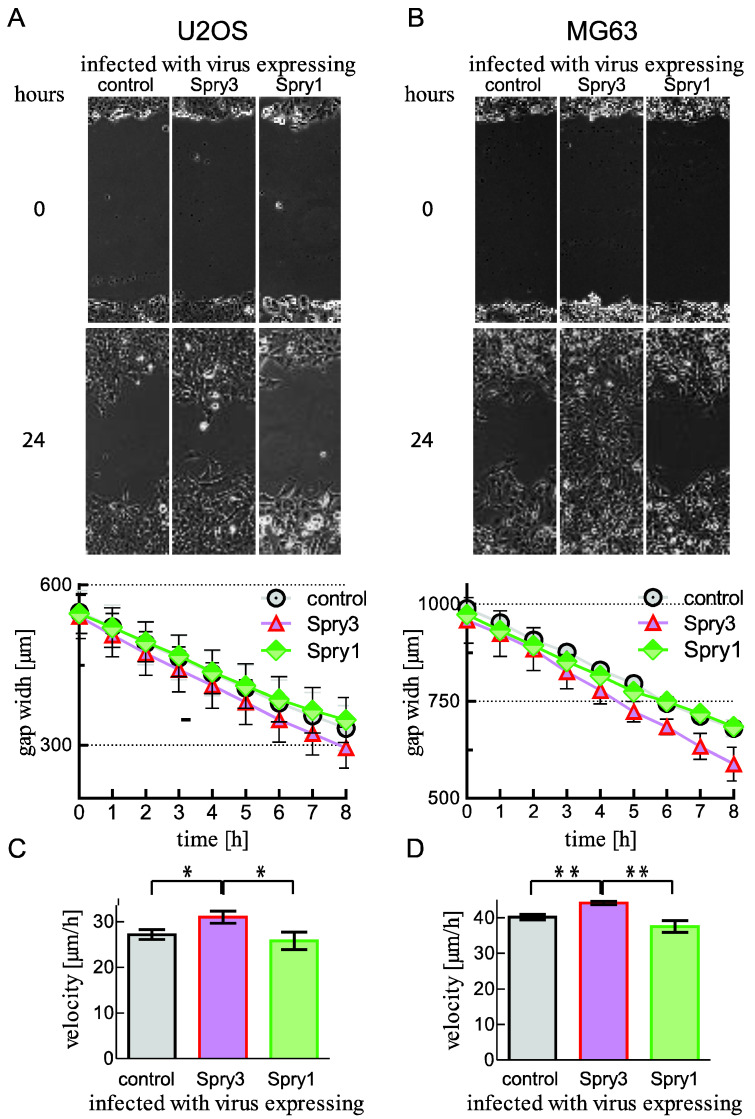
Impact of Spry3 and Spry1 on cell migration in osteosarcoma-derived cells. U2OS (**A**) and MG63 (**B**) cells expressing the indicated proteins were cultured to form a close layer before a scratch assay was performed. Representative pictures taken with a VISITRON Live Cell Imaging System at time points 0 and 24 h are presented. Measurements of three replicative gaps were performed every hour over a time period of eight hours and a representative experiment is depicted. Velocities of four experiments performed in U2OS (**C**) and MG63 (**D**) were calculated using linear regression in GraphPad Prism. The calculated speeds (µm/h) of the experiments are depicted as means ± SEM. Using an unpaired t-test, significance was determined. ** p < 0.01; *p < 0.05.

Concomitant with cell proliferation, molecules of signaling pathways usually affect cell migration in tumor cells. Therefore, we investigated how ectopic Spry3 and Spry1 expressions influence the velocity of gap closure in a Scratch assay using osteosarcoma-derived cells. Over a time period of 24 h the scratch is almost closed if the U2OS cells express Spry3 protein. In parallel, control treated as well as Spry1 expressing cells migrate clearly slower (Figure 4A), and the residual gap is still wide open (Figure 4A). In accordance, we observed that Spry3 expressing MG63 cells completely close the gap within 24 h, while in parallel, the gap in the control as well as the Spry1 protein expressing MG63 lawn was obviously still open (Figure 4B). Linear regression revealed that Spry3 overexpressing U2OS cells migrate with a velocity of 31 ± 1.3 µm/h, while cells treated with a control (27 ± 1.1 µm/h) or Spry1 coding virus (26 ± 1.9 µm/h) were significantly slower (Figure 4C).

Like in U2OS cells, in MG63 cells the velocity of gap closure was significantly increased in the presence of Spry3 (Figure 4D).

These studies corroborate the initial observation that in osteosarcoma derived cells Spry3 promotes tumor-associated features, while Spry1 expression has no effect.

### 2.5. Anchorage-Independent Growth of the Osteosarcoma-Derived Cell Line U2OS Is Facilitated by Increased Spry3 Levels

While operative removal and chemotherapeutic treatment are effective to fight the primary osteosarcoma, the formation of metastasis is the main cause for a deadly progression of this disease. In order to metastasize, cancer cells need to gain the ability to proliferate and survive anchorage independently. Since colony formation in a semisolid medium is shown to strongly associate with the metastatic potential of a tumor [35], we chose this in vitro method to get an indication for the influence of the Spry proteins on this progression stage of the tumor. To test the influence of Spry3 expression on anchorage-independent growth, a soft agar assay was performed with U2OS as well as MG63 cells incubated with adenoviruses expressing control, Spry3 or Spry1 protein, respectively.

**Figure 5:**
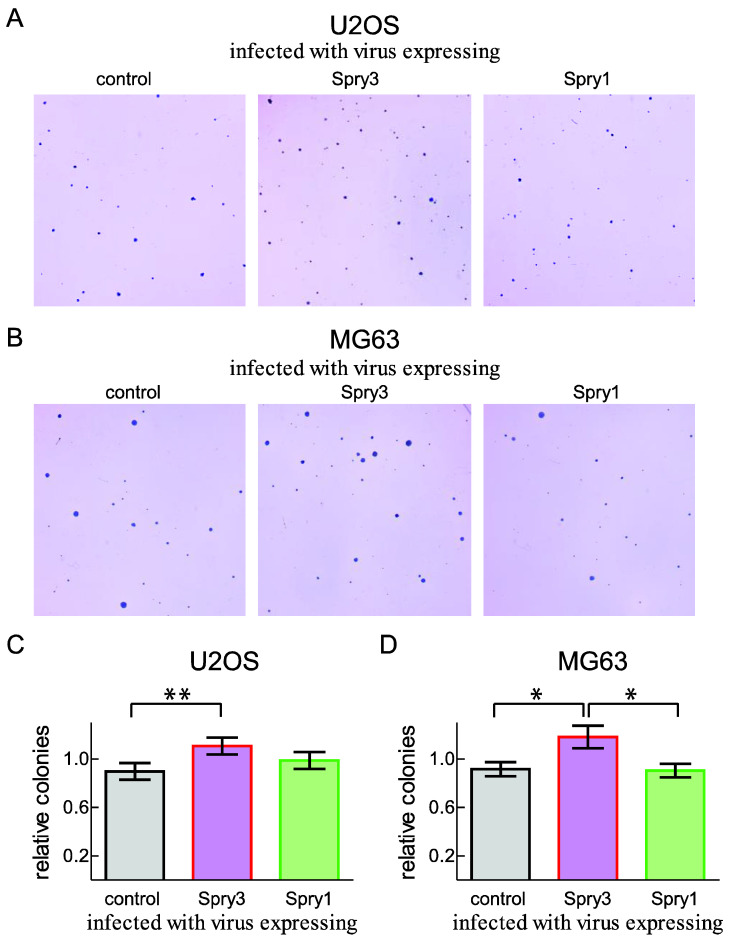
Influence of Spry3 and Spry1 expression on the ability of osteosarcoma-derived cells to form colonies in a semisolid medium. U2OS (**A**) and MG63 (**B**) cells were infected with adenoviruses expressing the indicated proteins. A representative section of a Soft agar plate is depicted. The U2OS (**C**) and MG63 (**D**) cell clones were counted and evaluated. To adjust the experimental variations, the obtained numbers of each experiment are given as the ratio to the experimental mean. Significance was assessed using an unpaired t-test in GraphPad Prism and means ± SEM are shown. ** *p* < 0.01; * p < 0.05.

As depicted in Figure 5A, U2OS cells are able to form colonies in soft agar, and if the cells are overexpressing Spry3, more and partly bigger colonies can be observed. Consistent with this observation, clone formation in MG63 was significantly improved in case of enforced Spry3 expression (Figure 5B). Evaluation of several independent assays revealed that in contrast to Spry1, Spry3 can endow U2OS (Figure 5C) and MG63 (Figure 5D) with capacities facilitating the colony formation in semisolid medium.

These results corroborate the conclusion of a tumor promoting potential of Spry3 in osteosarcoma-derived cells.

### 2.6. Spry3 Levels Are Beneficial for ERK Phosphorylation

**Figure 6:**
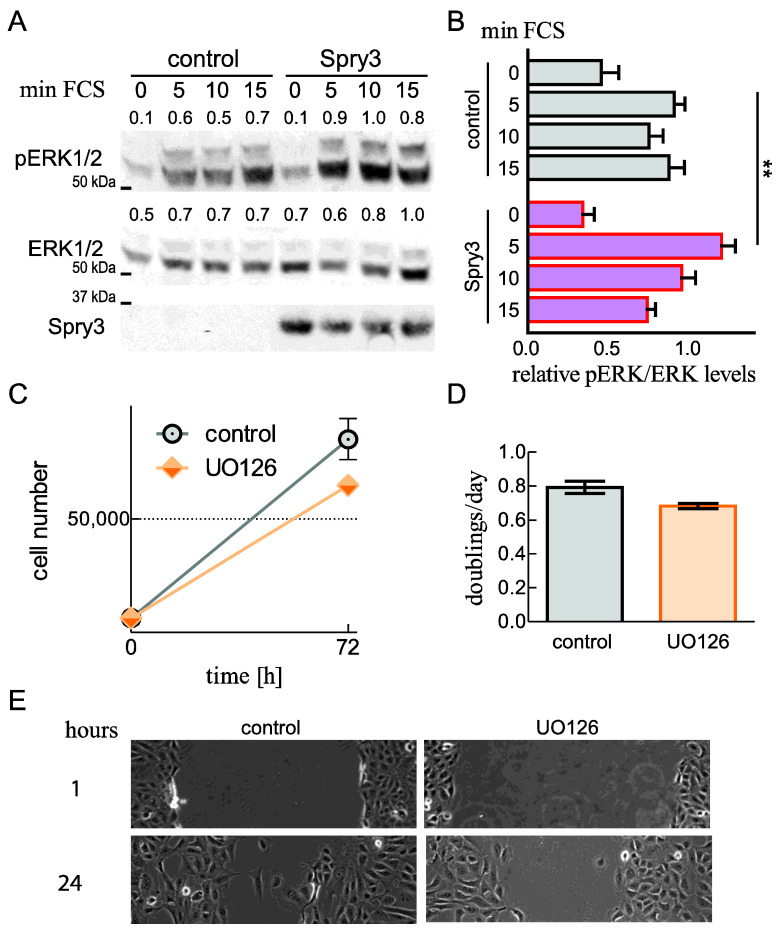
Influence of Spry3 protein on ERK activation. U2OS cells were serum-starved for 24 h and then infected with adenoviruses encoding either a control protein (luciferase) or Spry3 protein. After two more days, cells were incubated with serum for the indicated times. (**A**) Immunoblots were consecutively probed with antibodies recognizing total ERK1/2, pERK1/2 and Spry3. A representative experiment is pictured. Using ImageQuant 5.0, the intensity of the pERK1/2 and ERK1/2 bands were quantified and indicated. The highest values were arbitrarily set as 1. (**B**) The obtained values of pERK were normalized to the corresponding values calculated for the ERK expressions. A summary of calculated mean values ± SEM of the pERK/ERK ratios from three experiments is depicted. Significance between the three groups was calculated by using a one-way ANOVA test in GraphPad Prism. ** p < 0.01; (**C**) After plating, U2OS cells were counted, treated with DMSO (control) or the indicated inhibitor, and counted 72 h later. The numbers are depicted as a growth curve. (**D**) Doublings per day of replicates were calculated. (**E**) Microscopic pictures with 10× magnification showing gap closure of control as well as MEK inhibitor treated U2OS cells within a period of 24 h are depicted.

Although Spry proteins fulfill many roles in the fine tuning of signal transduction in different cells, in most reports their action is visible as modulated MAPK signaling. In order to investigate if Spry3 is able to influence this signaling pathway, we performed a cell signaling assay and monitored the ERK1/2 phosphorylation. In serum-starved U2OS cells, there is hardly phosphorylated ERK1/2 (pERK) visible, but after serum addition the signal is immediately augmented and stays high throughout the studied time period (Figure 5A). If cells ectopically express Spry3 instead of a control protein, the signal intensity peaks also immediately and the increase is more pronounced (Figure 6A). The augmentation of the relative amount of pERK due to Spry3 expression is reproducible and significantly detectable at the five min time point. After this immediate response the pERK levels drop in their intensities and are similar to the ones observed in control cells (Figure 6B). These data demonstrate that Spry3 is able to augment ERK phosphorylation and indicate that the Spry3-associated intensification of signal transduction via the MAPK pathway can be responsible for the observed acceleration of cell proliferation and migration. Accordingly, interference with MAPK activation using the MEK inhibitor UO126 decelerates cell proliferation as measured in a proliferation experiment (Figure 6C,D) and by using a scratch assay (Figure 6E). Nonetheless, the inhibitor fails to completely suppress these two processes (Figure 6C,E).

These data indicate that intensified activation of the MAPK pathway in the presence of Spry3 can cause increased proliferation and migration of U2OS cells.

### 2.7. Mutation of the Tyrosine in the N-Terminal Homologous Box of Spry3 Creates a Dominant-Negative Protein with an Inhibitory Role on Cell Migration

**Figure 7:**
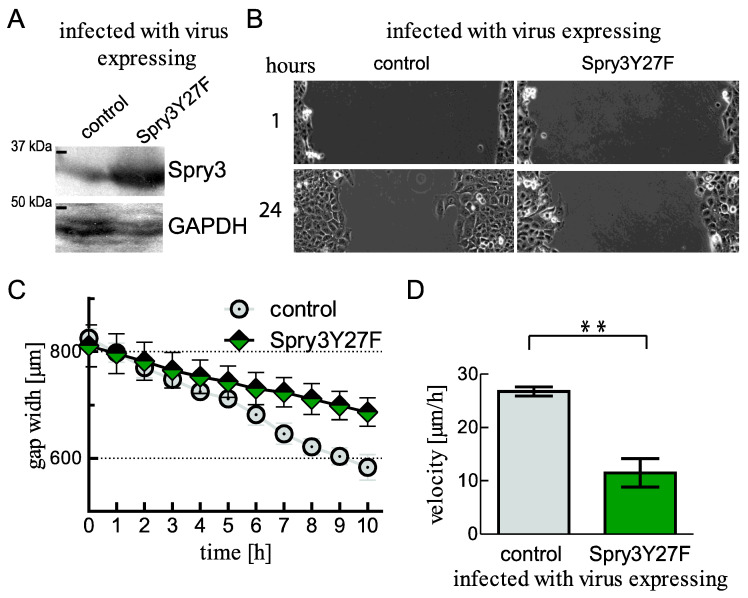
Influence of the mutated Spry3Y27F protein on cell migration. (**A**) U2OS cells were infected with adenoviruses expressing either a control or the mutated Spry3Y27F protein and expression of the introduced Spry3Y27F protein was proved by an immunoblot using antibodies recognizing Spry3. (**B**) A scratch assay with the cells expressing the indicated proteins was performed. Pictures of a representative scratch showing the gap at 1 and 24 h are shown. The loading was controlled by detecting GAPDH. (**C**) The distance coverage of three replicates of a representative scratch is depicted over the first 10 h. (**D**) By performing linear regression of the gap closure, the velocity was determined and the graph summarizes three experiments. Means ± SEM are shown. ** p < 0.01.

In case of Spry2 [36] and Spry4 proteins [37], it was reported that phosphorylation of a tyrosine within a homologous N-terminal box is crucial for Spry protein function, and the mutation of this conserved tyrosine results in an inactive or even dominant-negative form of the protein. To investigate if the tumor-promoting functions of Spry3 are dependent on the functional regulations conferred by this N-terminal box, we substituted the equivalent tyrosine at position 27 in Spry3 with a phenylalanine. Using the adenoviral system, the altered Spry3Y27F was expressed (Figure 7A) and a scratch assay was performed. As depicted in Figure 7B, after 24 h control-treated U2OS have already half-finished the gap closure, while in U2OS expressing a mutated Spry3 protein the gap width is only slightly altered at this time point. Calculation of migration velocity revealed that the average speed of U2OS (27 ± 0.9 µm/h) was slowed down to less than 12 µm/h (11.5 ± 2.7 µm/h) if the cells expressed the mutated version of Spry3 (Figure 7C,D).

These observations indicate that the N-terminal tyrosine is crucial for Spry3 function and its substitution creates a dominant-negative version, at least with respect to the migration-promoting action of Spry3.

### 2.8. Spry3 Expression Is Elevated in Tumor-Derived Cells

**Figure 8:**
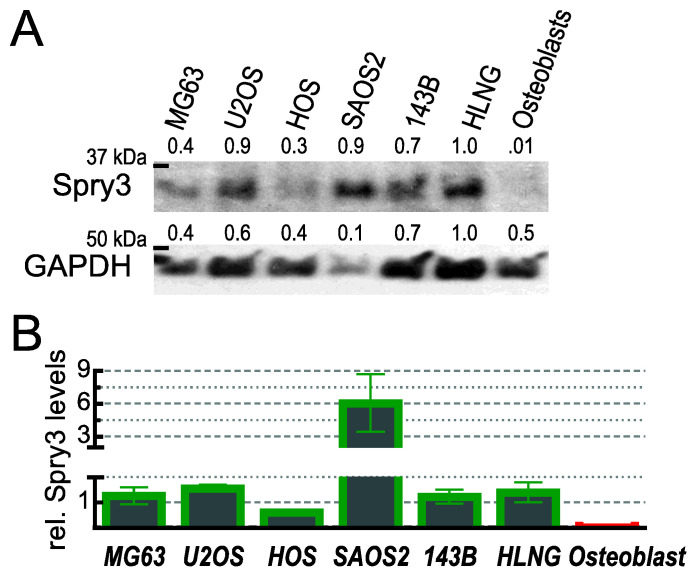
Expression of Spry3 protein in osteoblasts compared to osteosarcoma-derived cell lines. (**A**). Logarithmically growing cell lines derived from osteosarcoma- derived cell lines and from primary human osteoblasts were analyzed by an immunoblot sequentially probed with Spry3 and GAPDH. (**B**) Amounts of Spry3 proteins were measured by Image Quant software and normalized to GAPDH. The highest value was arbitrarily set as 1. Quantification results of two Western blots were depicted as mean ± SEM are shown in a column graph.

Apart their ability to facilitate tumor promoting characteristics of cells visible as enhanced cell migration, proliferation and anchorage-independent growth, a typical feature of an oncogene is its increased expression in tumor cells. To compare the expression of Spry3 in osteosarcoma with their normal cellular counterpart, immunoblotting analyses of protein extracts derived from logarithmically growing osteoblasts and different osteosarcoma-derived cell lines were performed. As depicted in Figure 8A, Spry3 is hardly detectable in osteoblasts, while all osteosarcoma-derived cell lines show a prominent expression, although to a different extent. Densitometric analysis revealed that in the tumor-derived cells Spry3 expression increased at least tenfold (Figure 8B).

In combination with its promoting role on malignant processes in tumor cells these expression data confirm the oncogenic function of Spry3 in osteosarcoma.

## 3. Discussion

The presented data show that osteosarcoma-derived cells express prominent levels of the Spry3 protein. Earlier studies demonstrated that in contrast to Spry1, Spry2 and Spry4, which are ubiquitously expressed in all organs, Spry3 detection is limited to the brain and testis [7]. Subsequent reports verify an expression of Spry3 in cells of neuronal origin associated with a regulative function [38]. In malignant cells, Spry3 expression was not exclusively detected in the brain [30], since in accordance with our data in a recent publication, Spry3 was detected in Ewing’s sarcoma, another bone associated cancer [39].

Furthermore, we observed that osteosarcoma cells with increased Spry3 do not necessarily express high or low Spry1 protein, but interestingly, in comparison with an earlier report investigating Spry2 and Spry4 in osteosarcoma-derived cell lines, it was revealed that MG63 and 143B, the only cell lines with prominent Spry1 levels, also showed high levels of Spry2 and Spry4 [34]. These data indicate that mechanisms regulating endogenous levels of Spry3 and the other Spry proteins are different. The independence of the protein levels on mitogen-availability suggest that both mechanisms regulating Spry3 and Spry1 are not connected to the negative feedback loop observed in case of mitogen-induced transcription of Spry2 and Spry4 [40]. The only available data studying the expression of Spry3 in dependence of serum availability show that—like in osteosarcoma—the absence of serum has no effect on Spry3 levels in brain cancer derived cells [30]. In response of FGF, its levels were decreased in bovine granulosa cells [41]. Spry1 expression was observed to fluctuate in response to pERK and mitogen induction. While Ozaki et al. found its level increasing as an effect of pERK induction [42], other reports show a decreased expression of Spry1 if FGF was supplied [31]. In lung cancer derived cells, Spry1 levels were unaffected by mitogens [43].

Increased expression of Spry1 fails to influence the tested biological processes connected to malignancy of osteosarcoma. In other types of sarcoma, Spry1 effects are already reported. In accordance with our data, Schaaf et al. observed that modulation of Spry1 levels had no influence on proliferation of rhabdomyosarcoma if the Ras genes were wildtype, and only if the cells harbor an oncogenic Ras version Spry1 functions as a tumor promoter [31]. But while Ras mutations in osteosarcoma are rare events [44], in the embryonal subtype of rhabdomyosarcoma around 40% of the tumors harbor a mutated Ras version [31]. In contrast to these studies, Spry1 fulfills the criteria of a tumor suppressor in Ewing’s sarcoma. It is suppressed by EWS-FLI1, the product of the most common chromosomal translocation of this tumor entity, and its re-expression interferes with proliferation and migration of Ewing’s sarcoma-derived cells [39]. In carcinoma, the published data show that Spry1 is suppressive in breast [45], prostate [32], and thyroid [33].

Along with the observations concerning Spry1, the herein presented data clearly demonstrate that Spry3 fulfills a tumor promoting function in osteosarcoma. Increased expression of the protein accelerates cell proliferation in both of the tested cell lines suggesting that endogenous levels are not indicative for the observed phenomena. Additionally, Spry3 promotes the velocity of cell migration. Both processes are important for establishing a tumor and therefore we can conclude that the malignant potential of osteosarcoma is increased by elevated Spry3 expression. A similar function of Spry3 is already established in glioblastoma [30]. Since the metastatic potential of osteosarcoma is crucial for patient’ prognosis, we additionally investigated how Spry3 is influencing the ability of the cells to form colonies in a semisolid medium. The results of our studies revealed that high Spry3 levels enable more cells to grow anchorage-independently, corroborating that Spry3 expression is beneficial for the malignant phenotype of osteosarcoma. Concomitant with the tumor-promoting impact, Spry3 expression enhances the amplitude of pERK signaling in response to serum. Accordingly, the tumor-promoting activities of Spry1 in rhabdomyosarcoma were also attributed to its positive regulatory functions within this pathway [31]. Modulation of the pathways targeting ERK phosphorylation is the most widely accepted function of Spry proteins, although in many studies reporting oncogenic functions the causal alteration was not detected within this cascades. In glioblastoma, for example, reduced signaling resulting in diminished pERK phosphorylation was observed as a result of Spry4 expression, but Spry3 exerted tumor-promoting activities without influencing these pathways [30]. In contrast, the antitumoral effect achieved by Spry2 silencing in glioblastoma is based on excessive activation of ERK signaling [27]. In colon neoplasia, Spry2 is considered as oncogene functioning via activating expression of the c-Met-receptor [25] and/or ZEB1 [46]. The mode of action hold responsible for this receptor upregulation is the sequestration of the E3 ligase cCbl, which is involved in the ubiquitination and subsequent degradation of many receptors [47]. Spry proteins are shown to bind this protein via the N-terminal box [19]. Although an interaction of Spry3 with cCbl is not explicitly documented, interference with the degradation of a receptor could be another mode of action explaining the tumor-promoting activity of Spry3. Furthermore, it is known that Spry2 exerts a strong tumor-suppressing activity in osteosarcoma, and Spry3 could interfere with this action. Since homo- and hetero-oligomerization of Spry proteins is possible [48], Spry3 might also function via forming a possible inactive heterodimer with the endogenous Spry2 protein.

If a Spry3 protein is mutated to a version where the tyrosine in the N-terminal box is substituted by a phenylalanine mimicking a phosphorylation negative protein, its ectopic expression is able to inhibit cell migration of the osteosarcoma-derived U2OS cells. This observation is corroborating the data leading to the conclusion that Spry3 fulfills oncogenic functions in osteosarcoma. Mutations of the tyrosine in this conserved N-terminal box of Spry proteins are in many aspects working dominant negative [37], although the Spry functions might be lost only partially [22,49].

Corroborating the ability to fulfill a tumor-promoting function in osteosarcoma, Spry3 proteins are heavily overexpressed in this bone tumor-derived cancer, while it is hardly detectable in normal human osteoblasts. This is in agreement with prior observations that Spry3 is primarily detected in brain and testis tissue [7]. Earlier, an increase of expression of this Spry form coinciding with the malignancy of the cancer cells was observed in brain associated tumors [30].

In summary the reported data demonstrate that Spry1 expression fails to significantly influence the proliferation, migration, and colony forming capacity of osteosarcoma, while increased Sprouty3 expression is beneficial for osteosarcoma in order to execute the malignant phenotype. Together with earlier data, now the influence of all Spry family members is investigated in osteosarcoma. Along with an inhibitory effect on proliferation and differentiation of osteoblasts [50,51,52], Spry2 decelerates proliferation and migration of osteosarcoma [34]. Like in case of Spry1, Spry4 expression caused no significant alteration of these processes [34].

## 4. Materials and Methods

### 4.1. Cell Lines

The used cell lines MG63, 143B, U2OS, SAOS2, and HOS were derived from human osteosarcoma of juvenile patients and were purchased from American Type Culture Collection (ATCC, Rockville, MD USA). Osteoblasts were ordered from Pelobiotech GmbH, Planegg, Germany (Nr O-GRO-001-500) and used between passages three and eight. Cells were cultured in Dulbecco’s Modified Eagle Medium (DMEM) containing 10% fetal calf serum (FCS), penicillin (100 U/mL) and streptomycin (100 µg/mL) at 37 °C in 7.5% CO_2_. Cells were kept no longer than two months. For experiments using MEK inhibitors, UO126 (10 μM; Cell Signaling Technology, Danvers, MA, USA) was added.

### 4.2. Adenoviral Infection of Cells

Ectopic protein expression was achieved by adenoviral infection. Adenoviruses expressing Spry3 [30] and luciferase [53] were already available. To generate a Spry1 expressing virus, the coding sequence of Spry1 was amplified from a healthy donor cDNA by PCR using the primers 5′-TAGCGAATTCGGATCCATGGATCCCCAA-3′ and 5′–TAGCGAATTCCTCGAGTCATGATGGTTTA-3′ to introduce EcoR1 sites enabling a subsequent cloning into the EcoR1 site of a pADlox plasmid. To construct the Spry3Y27F expressing virus, site-directed-mutagenesis using the sense primer 5′-TTCGTGGAACGGCCGCCAGCCCCCTGT-3′ and antisense oligonucleotide 5′-CGGCCGTTCCACGAAGTCATTGCTAGC-3′ was performed as described [22]. The obtained pADlox constructs were verified by sequencing (Microsynth, Balgach, Switzerland) and the adenoviruses were generated as described [54].

The concentration of virus preparations optimal for each cell line was calculated according to the OD_260_ as a ratio to the cyan fluorescence protein expressing virus necessary to infect at least 90% of the cells.

### 4.3. Immunoblotting

Protein isolation and the consecutive Immunoblotting were performed according to an established protocol [55]. The antisera recognizing Spry3 [30] and Spry1 [55] were produced and evaluated earlier. Loading was routinely verified with an antibody against GAPDH (sc-365062), which was like the antibodies recognizing ERK (sc-514302) obtained from Santa Cruz (Santa Cruz Biotechnology, Inc., Dallas, TX, USA). Antibodies generated against pERK (#9101) were ordered from Cell Signaling Technology (Danvers, MA, USA) and the secondary antibodies are sold by GE Healthcare (Chalfont St. Giles, UK).

### 4.4. Growth Curve

Growth curve analyses were conducted and results evaluated as described [34]. Each experiment was performed at least three times in triplicates.

### 4.5. Scratch Assay

Analogous to Celik-Selvi et al. [30] a Scratch assay using 5 × 10^5^ MG63 or 6 × 10^5^ U2OS cells was performed.

### 4.6. Soft Agar Colony Formation Assay

24 h prior testing, cells were infected with adenoviruses expressing the respective proteins and a bottom layer of 0.4% soft agar mixed with penicillin/streptomycin (P/S) was poured. Next day, 5 × 10^3^ infected cells were mixed with warm (42 °C) soft agar containing 10× DMEM, FCS, P/S and NaHCO_3_ to obtain a 0.3% top layer and placed onto the bottom layer dishes, which have prior been incubated at 37 °C for 30 min. The plates were incubated at 37 °C in 7.5% CO_2_ for three weeks. Every three days 500 µL of fresh growth medium was supplied. Colonies formed in the agar were visualized by staining with crystal violet (0.005% crystal violet solved in 3.7% formaldehyde, 0.14 mol/L NaCl, 0.03 mol/L KH_2_PO_4_ and 0.04 mol/L K_2_HPO_4_ × 3H_2_O). After two washing steps using H_2_O for 1 h, the visible clones were counted and categorized. Big colonies were scored as 5, middle ones as 3 and small ones as 1. To correct experimental variations, the values were then calculated as ratio of the mean value of each experiment.

### 4.7. Cell Signaling Assay

For determining the induction of MAPK pathway, 10^5^ cells were treated as described [56] by using 20% FCS.

## Data Availability

All data are included in the article.

## References

[1] Mirabello L., Troisi R.J., Savage S.A. (2009). Osteosarcoma incidence and survival rates from 1973 to 2004: Data from the surveillance, epidemiology, and end results program. Cancer.

[2] Mirabello L., Troisi R.J., Savage S.A. (2009). International osteosarcoma incidence patterns in children and adolescents, middle ages and elderly persons. Int. J. Cancer.

[3] Bishop M.W., Janeway K.A., Gorlick R. (2016). Future directions in the treatment of osteosarcoma. Curr. Opin. Pediatr..

[4] Morrow J.J., Khanna C. (2015). Osteosarcoma genetics and epigenetics: Emerging biology and candidate therapies. Crit. Rev. Oncog..

[5] Hubbard S.R., Miller W.T. (2007). Receptor tyrosine kinases: Mechanisms of activation and signaling. Curr. Opin. Cell Biol..

[6] Freeman M. (2000). Feedback control of intercellular signalling in development. Nature.

[7] Minowada G., Jarvis L.A., Chi C.L., Neubuser A., Sun X., Hacohen N., Krasnow M.A., Martin G.R. (1999). Vertebrate sprouty genes are induced by fgf signaling and can cause chondrodysplasia when overexpressed. Development.

[8] Taniguchi K., Ayada T., Ichiyama K., Kohno R., Yonemitsu Y., Minami Y., Kikuchi A., Maehara Y., Yoshimura A. (2007). Sprouty2 and sprouty4 are essential for embryonic morphogenesis and regulation of fgf signaling. Biochem. Biophys. Res. Commun..

[9] Basson M.A., Akbulut S., Watson-Johnson J., Simon R., Carroll T.J., Shakya R., Gross I., Martin G.R., Lufkin T., McMahon A.P. (2005). Sprouty1 is a critical regulator of gdnf/ret-mediated kidney induction. Dev. Cell.

[10] Basson M.A., Watson-Johnson J., Shakya R., Akbulut S., Hyink D., Costantini F.D., Wilson P.D., Mason I.J., Licht J.D. (2006). Branching morphogenesis of the ureteric epithelium during kidney development is coordinated by the opposing functions of gdnf and sprouty1. Dev. Biol..

[11] Shim K., Minowada G., Coling D.E., Martin G.R. (2005). Sprouty2, a mouse deafness gene, regulates cell fate decisions in the auditory sensory epithelium by antagonizing fgf signaling. Dev. Cell.

[12] Taketomi T., Yoshiga D., Taniguchi K., Kobayashi T., Nonami A., Kato R., Sasaki M., Sasaki A., Ishibashi H., Moriyama M. (2005). Loss of mammalian sprouty2 leads to enteric neuronal hyperplasia and esophageal achalasia. Nat. Neurosci..

[13] Gross I., Morrison D.J., Hyink D.P., Georgas K., English M.A., Mericskay M., Hosono S., Sassoon D., Wilson P.D., Little M. (2003). The receptor tyrosine kinase regulator sprouty1 is a target of the tumor suppressor wt1 and important for kidney development. J. Biol. Chem..

[14] Hanafusa H., Torii S., Yasunaga T., Nishida E. (2002). Sprouty1 and sprouty2 provide a control mechanism for the ras/mapk signalling pathway. Nat. Cell Biol..

[15] Sasaki A., Taketomi T., Kato R., Saeki K., Nonami A., Sasaki M., Kuriyama M., Saito N., Shibuya M., Yoshimura A. (2003). Mammalian sprouty4 suppresses ras-independent erk activation by binding to raf1. Nat. Cell Biol..

[16] Edwin F., Singh R., Endersby R., Baker S.J., Patel T.B. (2006). The tumor suppressor pten is necessary for human sprouty 2-mediated inhibition of cell proliferation. J. Biol. Chem..

[17] Akbulut S., Reddi A.L., Aggarwal P., Ambardekar C., Canciani B., Kim M.K., Hix L., Vilimas T., Mason J., Basson M.A. (2010). Sprouty proteins inhibit receptor-mediated activation of phosphatidylinositol-specific phospholipase c. Mol. Biol. Cell.

[18] Kim H.J., Bar-Sagi D. (2004). Modulation of signalling by sprouty: A developing story. Nat. Rev. Mol. Cell Biol..

[19] Wong E.S., Lim J., Low B.C., Chen Q., Guy G.R. (2001). Evidence for direct interaction between sprouty and cbl. J. Biol. Chem..

[20] Masoumi-Moghaddam S., Amini A., Morris D.L. (2014). The developing story of sprouty and cancer. Cancer Metastasis Rev..

[21] Shaw A.T., Meissner A., Dowdle J.A., Crowley D., Magendantz M., Ouyang C., Parisi T., Rajagopal J., Blank L.J., Bronson R.T. (2007). Sprouty-2 regulates oncogenic k-ras in lung development and tumorigenesis. Genes Dev..

[22] Sutterluty H., Mayer C.E., Setinek U., Attems J., Ovtcharov S., Mikula M., Mikulits W., Micksche M., Berger W. (2007). Down-regulation of sprouty2 in non-small cell lung cancer contributes to tumor malignancy via extracellular signal-regulated kinase pathway-dependent and -independent mechanisms. Mol. Cancer Res..

[23] Lo T.L., Yusoff P., Fong C.W., Guo K., McCaw B.J., Phillips W.A., Yang H., Wong E.S., Leong H.F., Zeng Q. (2004). The ras/mitogen-activated protein kinase pathway inhibitor and likely tumor suppressor proteins, sprouty 1 and sprouty 2 are deregulated in breast cancer. Cancer Res..

[24] Fong C.W., Chua M.S., McKie A.B., Ling S.H., Mason V., Li R., Yusoff P., Lo T.L., Leung H.Y., So S.K. (2006). Sprouty 2, an inhibitor of mitogen-activated protein kinase signaling, is down-regulated in hepatocellular carcinoma. Cancer Res..

[25] Holgren C., Dougherty U., Edwin F., Cerasi D., Taylor I., Fichera A., Joseph L., Bissonnette M., Khare S. (2010). Sprouty-2 controls c-met expression and metastatic potential of colon cancer cells: Sprouty/c-met upregulation in human colonic adenocarcinomas. Oncogene.

[26] Walsh A.M., Kapoor G.S., Buonato J.M., Mathew L.K., Bi Y., Davuluri R.V., Martinez-Lage M., Simon M.C., O’Rourke D.M., Lazzara M.J. (2015). Sprouty2 drives drug resistance and proliferation in glioblastoma. Mol. Cancer Res..

[27] Park J.W., Wollmann G., Urbiola C., Fogli B., Florio T., Geley S., Klimaschewski L. (2018). Sprouty2 enhances the tumorigenic potential of glioblastoma cells. Neuro Oncol..

[28] Tennis M.A., Van Scoyk M.M., Freeman S.V., Vandervest K.M., Nemenoff R.A., Winn R.A. (2010). Sprouty-4 inhibits transformed cell growth, migration and invasion, and epithelial-mesenchymal transition, and is regulated by wnt7a through ppargamma in non-small cell lung cancer. Mol. Cancer Res..

[29] Vanas V., Muhlbacher E., Kral R., Sutterluty-Fall H. (2014). Sprouty4 interferes with cell proliferation and migration of breast cancer-derived cell lines. Tumour Biol..

[30] Celik-Selvi B.E., Stutz A., Mayer C.E., Salhi J., Siegwart G., Sutterluty H. (2019). Sprouty3 and sprouty4, two members of a family known to inhibit fgf-mediated signaling, exert opposing roles on proliferation and migration of glioblastoma-derived cells. Cells.

[31] Schaaf G., Hamdi M., Zwijnenburg D., Lakeman A., Geerts D., Versteeg R., Kool M. (2010). Silencing of spry1 triggers complete regression of rhabdomyosarcoma tumors carrying a mutated ras gene. Cancer Res..

[32] Kwabi-Addo B., Wang J., Erdem H., Vaid A., Castro P., Ayala G., Ittmann M. (2004). The expression of sprouty1, an inhibitor of fibroblast growth factor signal transduction, is decreased in human prostate cancer. Cancer Res..

[33] Macia A., Gallel P., Vaquero M., Gou-Fabregas M., Santacana M., Maliszewska A., Robledo M., Gardiner J.R., Basson M.A., Matias-Guiu X. (2012). Sprouty1 is a candidate tumor-suppressor gene in medullary thyroid carcinoma. Oncogene.

[34] Rathmanner N., Haigl B., Vanas V., Doriguzzi A., Gsur A., Sutterluty-Fall H. (2013). Sprouty2 but not sprouty4 is a potent inhibitor of cell proliferation and migration of osteosarcoma cells. FEBS Lett..

[35] Mori S., Chang J.T., Andrechek E.R., Matsumura N., Baba T., Yao G., Kim J.W., Gatza M., Murphy S., Nevins J.R. (2009). Anchorage-independent cell growth signature identifies tumors with metastatic potential. Oncogene.

[36] Mason J.M., Morrison D.J., Bassit B., Dimri M., Band H., Licht J.D., Gross I. (2004). Tyrosine phosphorylation of sprouty proteins regulates their ability to inhibit growth factor signaling: A dual feedback loop. Mol. Biol. Cell.

[37] Sasaki A., Taketomi T., Wakioka T., Kato R., Yoshimura A. (2001). Identification of a dominant negative mutant of sprouty that potentiates fibroblast growth factor- but not epidermal growth factor-induced erk activation. J. Biol. Chem..

[38] Ning Z., McLellan A.S., Ball M., Wynne F., O’Neill C., Mills W., Quinn J.P., Kleinjan D.A., Anney R.J., Carmody R.J. (2015). Regulation of spry3 by x chromosome and par2-linked promoters in an autism susceptibility region. Hum. Mol. Genet..

[39] Cidre-Aranaz F., Grunewald T.G., Surdez D., Garcia-Garcia L., Carlos Lazaro J., Kirchner T., Gonzalez-Gonzalez L., Sastre A., Garcia-Miguel P., Lopez-Perez S.E. (2017). Ews-fli1-mediated suppression of the ras-antagonist sprouty 1 (spry1) confers aggressiveness to ewing sarcoma. Oncogene.

[40] Mayer C.E., Haigl B., Jantscher F., Siegwart G., Grusch M., Berger W., Sutterluty H. (2010). Bimodal expression of sprouty2 during the cell cycle is mediated by phase-specific ras/mapk and c-cbl activities. Cell. Mol. Life Sci..

[41] Jiang Z., Price C.A. (2012). Differential actions of fibroblast growth factors on intracellular pathways and target gene expression in bovine ovarian granulosa cells. Reproduction.

[42] Ozaki K., Kadomoto R., Asato K., Tanimura S., Itoh N., Kohno M. (2001). Erk pathway positively regulates the expression of sprouty genes. Biochem. Biophys. Res. Commun..

[43] Kral R., Doriguzzi A., Mayer C.E., Krenbek D., Setinek U., Sutterluty-Fall H. (2016). Differential effects of variations at codon 106 on sprouty2 functions in lung cancer-derived cells. J. Cell Biochem..

[44] Prior I.A., Lewis P.D., Mattos C. (2012). A comprehensive survey of ras mutations in cancer. Cancer Res..

[45] Mekkawy A.H., Pourgholami M.H., Morris D.L. (2014). Human sprouty1 suppresses growth, migration, and invasion in human breast cancer cells. Tumour Biol..

[46] Barbachano A., Ordonez-Moran P., Garcia J.M., Sanchez A., Pereira F., Larriba M.J., Martinez N., Hernandez J., Landolfi S., Bonilla F. (2010). Sprouty-2 and e-cadherin regulate reciprocally and dictate colon cancer cell tumourigenicity. Oncogene.

[47] Wong E.S., Fong C.W., Lim J., Yusoff P., Low B.C., Langdon W.Y., Guy G.R. (2002). Sprouty2 attenuates epidermal growth factor receptor ubiquitylation and endocytosis, and consequently enhances ras/erk signalling. EMBO J..

[48] Ozaki K., Miyazaki S., Tanimura S., Kohno M. (2005). Efficient suppression of fgf-2-induced erk activation by the cooperative interaction among mammalian sprouty isoforms. J. Cell Sci..

[49] Vaquero M., Cuesta S., Anerillas C., Altes G., Ribera J., Basson M.A., Licht J.D., Egea J., Encinas M. (2019). Sprouty1 controls genitourinary development via its n-terminal tyrosine. J. Am. Soc. Nephrol..

[50] Sanui T., Tanaka U., Fukuda T., Toyoda K., Taketomi T., Atomura R., Yamamichi K., Nishimura F. (2015). Mutation of spry2 induces proliferation and differentiation of osteoblasts but inhibits proliferation of gingival epithelial cells. J. Cell Biochem..

[51] Taketomi T., Onimura T., Yoshiga D., Muratsu D., Sanui T., Fukuda T., Kusukawa J., Nakamura S. (2018). Sprouty2 is involved in the control of osteoblast proliferation and differentiation through the fgf and bmp signaling pathways. Cell Biol. Int..

[52] Yang X., Webster J.B., Kovalenko D., Nadeau R.J., Zubanova O., Chen P.Y., Friesel R. (2006). Sprouty genes are expressed in osteoblasts and inhibit fibroblast growth factor-mediated osteoblast responses. Calcif. Tissue Int..

[53] Vanas V., Haigl B., Stockhammer V., Sutterluty-Fall H. (2016). Microrna-21 increases proliferation and cisplatin sensitivity of osteosarcoma-derived cells. PLoS ONE.

[54] Sutterluty H., Chatelain E., Marti A., Wirbelauer C., Senften M., Muller U., Krek W. (1999). P45skp2 promotes p27kip1 degradation and induces s phase in quiescent cells. Nat. Cell Biol..

[55] Kral R.M., Mayer C.E., Vanas V., Gsur A., Sutterluty-Fall H. (2014). In non-small cell lung cancer mitogenic signaling leaves sprouty1 protein levels unaffected. Cell Biochem. Funct..

[56] Stutz A., Kamptner A.Z.M., Sutterluty H. (2021). A sprouty4 mutation identified in kallmann syndrome increases the inhibitory potency of the protein towards fgf and connected processes. Int. J. Mol. Sci..

